# Reference genes for reverse transcription quantitative PCR in canine brain tissue

**DOI:** 10.1186/s13104-015-1628-4

**Published:** 2015-12-09

**Authors:** Quirine E. M. Stassen, Frank M. Riemers, Hannah Reijmerink, Peter A. J. Leegwater, Louis C. Penning

**Affiliations:** Department of Clinical Sciences of Companion Animals, Faculty of Veterinary Medicine, Utrecht University, PO Box 80154, 3508 TD Utrecht, The Netherlands

**Keywords:** Reference genes, Dog, Brain, RT-qPCR

## Abstract

**Background:**

In the last decade canine models have been used extensively to study genetic causes of neurological disorders such as epilepsy and Alzheimer’s disease and unravel their pathophysiological pathways. Reverse transcription quantitative polymerase chain reaction is a sensitive and inexpensive method to study expression levels of genes involved in disease processes. Accurate normalisation with stably expressed so-called reference genes is crucial for reliable expression analysis.

**Results:**

Following the minimum information for publication of quantitative real-time PCR experiments precise guidelines, the expression of ten frequently used reference genes, namely *YWHAZ, HMBS, B2M, SDHA, GAPDH, HPRT, RPL13A, RPS5, RPS19* and *GUSB* was evaluated in seven brain regions (frontal lobe, parietal lobe, occipital lobe, temporal lobe, thalamus, hippocampus and cerebellum) and whole brain of healthy dogs. The stability of expression varied between different brain areas. Using the GeNorm and Normfinder software *HMBS*, *GAPDH* and *HPRT* were the most reliable reference genes for whole brain. Furthermore based on GeNorm calculations it was concluded that as little as two to three reference genes are sufficient to obtain reliable normalisation, irrespective the brain area.

**Conclusions:**

Our results amend/extend the limited previously published data on canine brain reference genes. Despite the excellent expression stability of *HMBS*, *GAPDH* and *HRPT*, the evaluation of expression stability of reference genes must be a standard and integral part of experimental design and subsequent data analysis.

**Electronic supplementary material:**

The online version of this article (doi:10.1186/s13104-015-1628-4) contains supplementary material, which is available to authorized users.

## Background

Companion dogs that cohabitate with their owners under similar environmental conditions frequently develop the same hereditary diseases as people do [1, 2]. For example aging dogs may demonstrate cognitive decline with distinctive neuropathological features that parallel early Alzheimer’s disease [3]. Likewise human and canine primary epilepsy share many clinical characteristics [4].

Recently Briggs et al. [5] found that the expression of orthologous genes between matched canine and human tissues was remarkably similar. Inbreeding in dogs narrows the genetic variation within breeds and works as a magnifier to dissect the genetic background of inherited diseases which is one of the reasons for an in-depth sequencing of the dog’s genome [6]. As a consequence there has been a keen interest in utilising the dog both as a pathophysiological and genetic model for common human diseases [1, 7]. This has resulted in the discovery of novel causative mutations in several disorders, among others in hereditary epilepsy [8, 9] and elucidation of the pathological pathways involved [10]. An example of the latter is the identification of the mutation in *HCRTR2* causing narcolepsy in Doberman Pinschers which led to the unravelling of a pathway involved in this disease and sleep regulation [11]. Canine brain models have been widely used to study epilepsy and Alzheimer’s disease [3, 4, 12, 13]. These studies are of great significance for both human and veterinary medicine.

Knowledge of gene expression is essential for understanding how genes regulate both physiological and pathological processes at a fundamental level. Reverse transcription in combination with quantitative polymerase chain reaction (RT-qPCR) has become the method of choice for quantifying mRNA expression in biological tissues because of its high sensitivity, accuracy and relatively low costs. During such a complex multi-step procedure experimental errors between samples (e.g., differences in initial sample RNA amounts, efficiency of RNA isolation, RNA integrity and efficiency of reverse transcription) can easily occur [14, 15]. To correct for these errors data normalisation is indispensable. The most popular normalisation strategy involves the use of stably expressed endogenous reference genes to which expression of the gene(s) of interest can be related. However expression of commonly used reference genes has been reported to vary between tissues, individuals, species, methods used and to be influenced by pathological conditions and therapies [16–18]. Dheda et al. [19] and Ohl et al. [20] demonstrated that the outcome of expression analysis is highly dependent on the reference gene chosen. Therefore selection of inappropriate reference genes or insufficient numbers of reference genes, resulting in erroneous conclusions, poses a major problem in expression studies.

To the best of the authors’ knowledge only two papers concerning identification of reference genes for the canine brain have been published [21, 22]. Unfortunately, the results of those studies didn’t correspond with each other. This emphasises the need to come up with a normalisation strategy in canine brain tissue that allows for the comparison of data and independent replication of experiments. The precise minimum information for publication of quantitative real-time PCR experiments (MIQE) guidelines describe such a strategy [23, 24]. Following these guidelines, the expression of ten frequently used reference genes in seven brain regions from seven healthy dogs was studied to determine which reference genes or combination there of could be used for valid data interpretation.

## Methods

### Tissue sampling

Normal brain tissue was obtained as surplus material from seven healthy dogs that were euthanised in non-related experiments approved by the Utrecht University Animal Experiments Committee as required under Dutch legislation and according to the University’s 3Rs-policy. The group included 4 mixed-breeds, 2 Labrador Retrievers and 1 Beagle; 4 males 3 females; age ranging from 4 months to 9 years.

The brain was removed and dissected immediately after death had occurred. Tissue samples from the cerebral cortex (frontal lobe, parietal lobe, occipital lobe and temporal lobe), thalamus, hippocampus and cerebellum were obtained from each dog and placed in 5–10 volumes RNA later^®^ stabilisation fluid (Ambion, Austin, TX) for 24–48 h. Subsequently supernatant was removed and samples were stored at −70 °C until assayed.

### Primer design

The following candidate reference genes, representing different functional classes, were selected: *YWHAZ* (coding for zeta polypeptide)*, HMBS* (for hydroxymethylbilane synthase)*, B2M* (for beta-2-microglobulin)*, SDHA* (for succinate dehydrogenase complex subunit A)*, GAPDH* (for glyceraldehyde-3-phosphate dehydrogenase), *HPRT* (for hypoxanthine guanine phosphoribosyl transferase)*, RPL13A* (for ribosomal protein 13A)*, RPS5* (for ribosomal protein S5)*, RPS19* (for ribosomal protein S19) and *GUSB* (for beta-glucuronidase).

Primer design was performed with Oligo Explorer 1.1.0 software [25]. Forward and reverse primers were positioned in different exons to reduce the chance of amplification of genomic DNA where feasible. Details of the primers including exonic locations are depicted in Additional file 1: Table S1. Basic local alignment search tool [26] searches were performed to verify specificity of each primer.

### RNA isolation, reverse transcription and quantitative PCR

Total RNA was isolated from RNA later fixed samples, using Qiagen RNeasy Mini Kit (Qiagen, Leusden, The Netherlands) according to the manufacturer’s instructions including an on-column deoxyribonulease I (DNase I) treatment. RNA quantity and quality were evaluated spectrophotometrically using Nanadrop ND-1000 (Isofen Life Sciences, IJsselstein, The Netherlands) and an Agilent BioAnalyzer 2100 (Agilent, Palo Alto, CA) respectively. All RNA integrity number values, based on 28S and 18S integrity, were above 7.0 (range 7.0–8.3) indicating good quality RNA. Reverse transcription was performed with 50 ng of total RNA in a total reaction volume of 20 μl using iScript™ cDNA Synthesis Kit (Bio-rad, Veenendaal, The Netherlands) containing a mix of both oligo-dT and random hexamer primers.

After cDNA synthesis the reactions were diluted tenfold and a part of each reaction was pooled and used in a fourfold serial dilution to assess the amplification efficiency of each gene. The rest of the reaction was diluted fivefold and used as a template to measure the gene expression in technical duplicates.

RNA samples were tested for contamination with genomic DNA by qPCR of non-reverse-transcribed RNA templates (minus RT-controls). Also a no template control was included containing ultrapure water instead of cDNA to test for reaction contaminants and primer dimer formation.

The qPCR reaction was performed on a MyiQ™ quantitative PCR machine (Bio Rad). Reactions contained 12.5 µl of 2× IQ SYBRGreen SuperMix (Bio Rad), 400 nM of each primer (Eurogentec), 1 µl cDNA template and ultrapure water to a reaction volume of 25 µl.

Cycling conditions were 3 min at 95 °C, followed by 45 cycles with denaturing template for 20 s at 95 °C, followed by 30 s at melting temperature (Tm), and elongation at 72 °C for 30 s. When the annealing temperature (Ta) was higher than 57 °C, the elongation step at 72 °C was omitted and extension took place at Ta.

Subsequently, a melt curve, to verify amplification of a single product, was generated starting at 65 °C and increasing by 1 °C every 30 s to 99 °C.

The amplification efficiency was always between 92 and 107 %. As the limit of detection (LOD) the Cq-value of the highest standard dilution was used and values for the measured genes were found to be: GUSB: 32.65, B2M: 27.18, RPL13: 25.99, RPS19: 27.94, RPS5: 26.89, SDHA: 26.79, YWHAZ: 25.83, GAPDH: 23.84, HMBS: 32.63, HPRT: 27.84. With regard to all the measured genes, none of the sample values fell below the LOD value of the measured gene.

The DNase treatment and primer locations minimised potential PCR artefacts caused by contaminating genomic DNA. Sequencing (Applied Biosystems, BigDye Terminator version 3.1) of the amplicon products confirmed product specificity.

### Data analysis

Data were analysed using GeNorm and NormFinder. Both freeware programs have unique properties; a combination of both analyses will produce reliable data. GeNorm calculates the stability of expression (M) of one gene based on the average pair-wise variation between all the studied reference genes. Stepwise elimination of the least stable gene finally identifies the two most stable genes [27]. Furthermore, GeNorm determines the optimal number of reference genes by calculating the normalisation factor (NF) for a given number of reference genes (n). Next the pairwise variation (V) between the consecutive normalisation factors NF_n_ and NF_n+1_ is defined. The lower V, the smaller the variation, implying that adding an extra gene doesn’t significantly improve normalisation. A cut-off value of 0.15 for the pairwise variation is most often chosen, indicating that the use of a set of reference genes with a pairwise variation <0.15 results in valid normalisation [14, 20, 24, 27, 28]. GeNorm analysis was performed using R (Version 2.12.0, Free Software Foundation) [29] and the GeNorm method from the SLqPCR package [30].

NormFinder analysis is a model-based approach, which calculates the overall variation in expression within sample groups of interest (intragroup variation) and the variation across the sample groups (intergroup variation) for each evaluated reference gene [31]. The combination of the two variation parameters results in a stability value, representing a practical measure of the systematic error that will be introduced when using a particular reference gene. NormFinder calculations were done using the NormFinder plug-in for Microsoft Excel [32].

## Results

The ten potential reference genes could be amplified in all brain regions evaluated. Both RT-minus and water controls were negative implying a lack of contaminating genomic DNA.

**Fig. 1:**
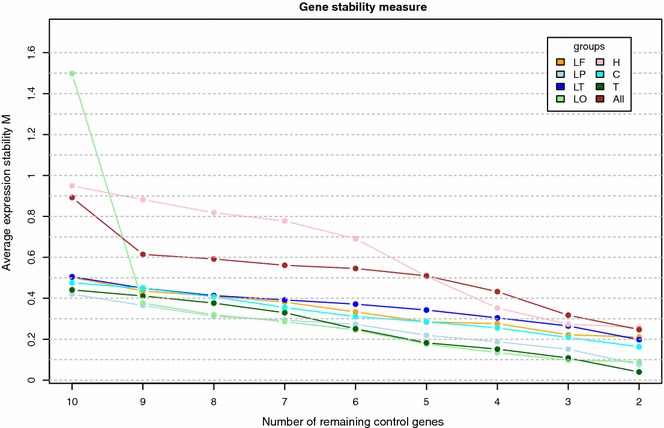
Average expression stability (M) of remaining reference genes during subsequent elimination of the least stable gene in different brain regions. M was calculated for all genes and the gene with the highest M-value was excluded in the next calculation round. Results are based on analysis of 7 brain areas of 7 dogs each: *LF* lobus frontalis, *LP* lobus parietalis, *LT* lobus temporalis, *LO* lobus occipitalis, *H* hippocampus, *C* cerebellum, *T* thalamus, *All* average of all areas

GeNorm analysis determined variable stability of reference genes in different brain areas (Table 1). The curves in Fig. 1 represent the average expression stability (M) of remaining reference genes during the step-by-step elimination of the least stable reference gene in different brain regions. A high M value indicates low expression stability. In most brain areas all calculated expression stabilities were below 1.0, indicating fairly stable expression of the reference genes evaluated. Expression stabilities (M) for the average of all evaluated areas (“whole brain”) were calculated and also plotted in Fig. 1. *GUSB* had the highest M-value (implying that it is the least stable gene) and was therefore excluded first. *HMBS* and *GAPDH* turned out to be the most stably expressed genes.

**Table 1:** GeNorm ranking of reference genes in order of their expression stability for specific brain regions

Frontal lobe	Parietal lobe	Temporal lobe	Occipital lobe	Hippocampus	Cerebellum	Thalamus	All
*HMBS*	*HMBS*	*RPL13A*	*HMBS*	*RPL13A*	*HMBS*	*GAPDH*	*HMBS*
*HPRT*	*YWHAZ*	*RPS5*	*HPRT*	*RPS19*	*SDHA*	*HPRT*	*GAPDH*
*GAPDH*	*SDHA*	*HPRT*	*SDHA*	*RPS5*	*YWHAZ*	*RPL13A*	*HPRT*
*RPS5*	*GUSB*	*GAPDH*	*B2M*	*B2M*	*GAPDH*	*RPS5*	*RPL13A*
*RPL13A*	*GAPDH*	*YWHAZ*	*YWHAZ*	*HMBS*	*B2M*	*RPS19*	*SDHA*
*RPS19*	*B2M*	*RPS19*	*GAPDH*	*GAPDH*	*GUSB*	*YWHAZ*	*RPS5*
*GUSB*	*RPS19*	*HMBS*	*RPL13A*	*SDHA*	*HPRT*	*SDHA*	*RPS19*
*SDHA*	*HPRT*	*SDHA*	*RPS19*	*HPRT*	*RPS19*	*HMBS*	*YWHAZ*
*YWHAZ*	*RPS5*	*GUSB*	*RPS5*	*GUSB*	*RPL13A*	*GUSB*	*B2M*
*B2M*	*RPL13A*	*B2M*	*GUSB*	*YWHAZ*	*RPS5*	*B2M*	*GUSB*

Decreasing from top to bottom; the most stable genes are depicted on top

*All* average of all areas

**Fig. 2:**
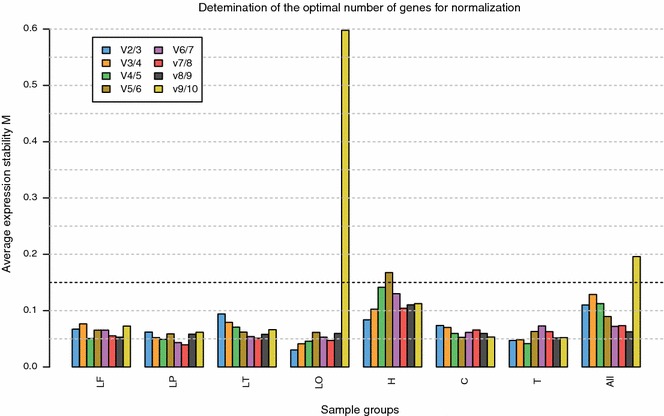
Determination of the optimum number of reference genes for normalisation of RT-qPCR data. The GeNorm program calculates the normalisation factor (NF) for a given number of genes (n) and defines the pairwise variation (V) between the sequential normalisation factors NF_n_ and NF_n+1_. For example *V2/3* represents the variation in NFs using the two versus three most stable control genes. A low V value indicates little variation, implying that adding the extra gene has no significant effect. Pairwise variation (V) < 0.15 is regarded to result in valid normalisation. Using this cut-off value all evaluated brain regions require only two reference genes for accurate normalisation. Results are based on analysis of 7 brain areas of 7 dogs each: *LF* lobus frontalis, *LP* lobus parietalis, *LT* lobus temporalis, *LO* lobus occipitalis, *H* hippocampus, *C* cerebellum, *T* thalamus, *All* average of all areas

The GeNorm program also calculated the minimum number of reference genes required for valid normalisation (Fig. 2). Based on a cut-off value of 0.15 for the pairwise variation only two reference genes are sufficient to obtain a reliable NF in all evaluated brain regions and the average of all areas.

According to the NormFinder program *HMBS* is the most stably expressed reference gene with a stability value of 0.005. *GAPDH, HPRT, RPS19* and *GUSB* share the second best stability value of 0.007. Therefor the best combination of two genes is *HMBS* with one of the above-mentioned genes resulting in a stability value of 0.004. These results correspond well with the results of the GeNorm analysis that identified *HMBS* and *GAPDH* as the two most stable control genes (Table 2).

**Table 2:** Ranking of reference genes based on GeNorm and NormFinder calculations, averages of all evaluated brain areas

Ranking order	GeNorm	NormFinder	NormFinder stability value
1 most stable	*HMBS*	*HMBS*	0.005
2	*GAPDH*	*GAPDH*	0.007
3	*HPRT*	*HPRT*	
4	*RPL13*	*RPS19*	
5	*SDHA*	*GUSB*	
6	*RPS5*	*RPS5*	0.008
7	*RPS19*	*B2M*	
8	*YWHAZ*	*RPL13*	0.009
9	*B2M*	*SDHA*	
10 least stable	*GUSB*	*YWHAZ*	0.025

A low stability value in NormFinder calculations corresponds with high expression stability. The GeNorm program doesn’t distinguish between the two most stably expressed genes (*HMBS* and *HRPT*) because gene ratios are required for the calculations

## Discussion

Gene expression analysis by RT-qPCR has become increasingly important in studies trying to disentangle the pathogenesis of different disease processes in various species. In 2006 and 2007 two papers were published evaluating various reference genes in a plethora of canine tissues [28, 33]. Unfortunately brain was not included in one of these. The first evaluation of reference gene expression stability in canine brain was presented by Vage et al. in which differential gene expression in brain tissue of aggressive and normal dogs was studied [21]. Because of the nature of their studies Vage et al. focussed on four brain areas known to be involved in behavioural reactions (amygdala, frontal cortex, hypothalamus and parietal cortex). To verify cDNA subtraction assays, qPCR was performed on nine differentially expressed genes and eight different reference genes (*ACTB, HNRNPH, HPRT1, RPL32, RPS5, TBP, TFRC* and *TKT*) were included. Based on GeNorm evaluation, *RPL32* and *HNRNPH1* expressions were selected for normalisation. Ranking of reference genes was not reported for the different anatomical regions. This paper is, apart from the specific set of differentially expressed genes, a landmark since it is one of the very few papers addressing the issue of gene expression normalisation in canine brain tissue.

Park et al. selected endogenous control genes for whole brain and different canine brain regions (forebrain divided in cerebrum and diencephalon, hindbrain including metencephalon) [22]. Results of RT-qPCR studies were analysed by GeNorm, NormFinder and BestKeeper software programs. However analyses were based on tissue samples from only two 12 month-old male Beagles, with most of the specific anatomical regions collected in only one dog. *RPS5* was found to be the most stably expressed control gene for whole brain tissue.

In the present research we studied expression of ten frequently used reference genes in seven healthy dogs of diverse breeds, both male and female in a wide age range (4 months to 9 years). Seven different brain regions (frontal lobe, parietal lobe, occipital lobe and temporal lobe, thalamus, hippocampus and cerebellum) relevant for brain disorders like epilepsy, Alzheimer’s disease, psychological and movement disorders were evaluated. “MIQE” guidelines [24] were followed to guarantee the technical quality of this research and allow assessment of experimental design. Data analysis was performed using two independent freeware platforms. Both GeNorm and NormFinder determined *HMBS* and the traditional reference gene *GAPDH* to be among the most stably expressed internal control genes. However expression stabilities of the seven genes next in rank are not far apart. This is demonstrated by the same or only marginal higher stability values in NormFinder calculations and the only gentle slope of the “all” curve from the point of nine remaining reference genes in Fig. 1. The GeNorm manual suggests a cut-off M-value of 1.5, indicating that genes with M-values lower than this limit are stable enough to be used as reference gene. M-values of the nine most stably expressed genes evaluated in this study were all well under this 1.5 limit, with *HMBS* and *GAPDH* reaching a value of 0.24. Likewise stability values of these genes calculated by NormFinder were low (range 0.005–0.009). The combination of two genes (*HMBS* and *GAPDH/HRTP/RPS19/GUSB*) further improved the stability value to 0.004. The GeNorm program calculated that using only two reference genes (*HMBS* and *GAPDH*) is sufficient for valid normalisation. However the minimum use of three stable control genes is recommended by the GeNorm programme developers [24, 27].

Although all candidate reference genes evaluated in the present study were included in the research of Park et al., results differed considerably. Park et al. reported *RPS5*, which was also included in the study of Vage et al., to be the most stable control gene for whole brain tissue. In our study it was assigned to the genes with relatively moderate stable expression. Conversely *HMBS* and *GAPDH* which we identified as the most stable genes were only average performers in the study by Park et al. *B2M* and *YWHAZ* showed relatively poor expression stability in both studies. Vage et al. identified *RPL32* and *HNRNPH1* as the most appropriate reference genes for canine brain tissue. Both genes were tested by Park et al. as well, but didn’t achieve top ranking. These inconsistencies may be caused by the various anatomical regions analysed, the small number of dogs in the study by Park et al. or different experimental settings. Another contributing factor may be that expression stabilities of some candidate control genes were found to be close to each other. As a result, only small variations in calculated expression stability can cause substantial changes in ranking order. This is illustrated by the fact that in our NormFinder calculations the genes ranked second to fifth all have the same stability value. As a consequence ranking is rather arbitrary.

Comparing studies on brain reference genes in humans, dogs, rats and mice is difficult because of different sets of candidate genes selected and variable experimental design [34–39]. Results vary between and sometimes within species and brain regions evaluated. In a cross-species reference gene evaluation in pituitary samples from humans, mice and dogs van Rijn et al. demonstrated that the expression stability varied between the three species [18].

Both Wang [38] and Johansson [34] paper identified *IPO8* and *POLR2A* as the two most stably expressed reference genes in human cerebral cortex (regions evaluated respectively parietal lobe and motor cortex) out of a large number of candidates (respectively 24 and 15). In Johansson paper brain cortices of chronic alcoholics and controls were included, whereas the research of Wang et al. evaluated brain tissue without pre-existing neurological pathology.

Together these papers clearly underline that the most stably expressed reference genes, and the number needed for accurate normalisation, vary among different experimental procedures, even if similar tissues of the same species are included. The importance of using validated transparent research protocols and appropriate internal control genes has been highlighted in several papers [14, 19, 24, 31]. Still numerous studies on RT-qPCR experiments using not validated and/or only one internal control gene have been published. A recent study analysed 1700 scientific papers that used RT-qPCR as a gene expression tool [40]. The vast majority of these 1700 papers did not report adequately on the details of the expression measurements. As little as around 10 % implemented the MIQE-guidelines. What’s most worrisome is that the number of reference genes included and the use of validated reference genes to normalise gene expression was negatively correlated with the journal’s impact factor [40].

## Conclusion

In order for RT-qPCR studies to be of clinical relevance it is of utmost importance that data are presented in a way that permits proper data comparison and experimental repetition. Furthermore accurate normalisation of data using stably expressed reference genes is indispensable. However expression of reference genes has been shown to vary among species, tissues and experimental settings. Therefore systematic reference gene stability evaluation must be an integral part of the experimental design.

Still the first step in identifying these reference genes is the selection of likely candidates. Wang [38] and the Johansson [34] papers prove that established tissue specific internal control genes can be very good candidates for other research projects on the same tissue. We conclude that *HMBS*, *GAPDH* and *HPRT* are excellent candidate reference genes for canine brain RT-qPCR studies.

## Additional files

10.1186/s13104-015-1628-4 Details of primers evaluated in this research: primer sequences, product sizes, exonic locations, optimal melting temperatures and GenBank accession numbers.

## Abbreviations

qPCR: quantitative polymerase chain reaction
RT-qPCR: reverse transcription quantitative polymerase chain reaction
MIQE: minimum information for publication of quantitative real-time PCR experiments
HCRTR2: hypocretin receptor 2
YWHAZ: zeta polypeptide
HMBS: hydroxymethylbilane synthase
B2M: beta-2-microglobulin
SDHA: succinate dehydrogenase complex subunit A
GAPDH: glyceraldehyde-3-phosphate dehydrogenase
HPRT: hypoxanthine guanine phosphoribosyl transferase
RPL13A: ribosomal protein 13A
RPS5: ribosomal protein S5
RPS19: ribosomal protein S19
GUSB: beta-glucuronidase
DNase I: deoxyribonuclease I
RIN: RNA integrity number
Ta: annealing temperature
ACTB: beta-actin
HNRNPH: heterogeneous nuclear ribonucleoprotein H
RPL32: ribosomal protein 32
TBP: TATA box binding protein
TFRC: transferrin receptor
TKT: transketolase
IPO8: importin 8
POLR2A: polymerase II polypeptide A
