# Supplementary material for: Reference genes for reverse transcription quantitative PCR in canine brain tissue
Source: BMC Res Notes. 2015 Dec 9;8:761. doi: 10.1186/s13104-015-1628-4 (PMC4673830; doi:10.1186/s13104-015-1628-4)
Supplement: Supplementary file 1 — 10.1186/s13104-015-1628-4 Details of primers evaluated in this research: primer sequences, product sizes, exonic locations, optimal melting temperatures and GenBank accession numbers. [file 13104_2015_1628_MOESM1_ESM.docx]

| Gene | Dir | Seq 5' -> 3' | Size (bp) | Exon | Temp | Accession |
| --- | --- | --- | --- | --- | --- | --- |
| *GAPDH* | U | TGTCCCCACCCCCAATGTATC | 100 | 2 | 58 | NM_001003142 |
|  | L | CTCCGATGCCTGCTTCACTACCTT |  | 2 |  |  |
| *HPRT* | U | AGCTTGCTGGTGAAAAGGAC | 104 | 5 and 6 | 56 | NM_001003357 |
|  | L | TTATAGTCAAGGGCATATCC |  | 7 |  |  |
| *RPS19* | U | CCTTCCTCAAAAAGTCTGGG | 95 | 2 and 3 | 61 | XM_533657 |
|  | L | GTTCTCATCGTAGGGAGCAAG |  | 3 |  |  |
| *GUSB* | U | AGACGCTTCCAAGTACCCC | 103 | 4 | 62 | NM_001003191 |
|  | L | AGGTGTGGTGTAGAGGAGCAC |  | 5 |  |  |
| *RPS5* | U | TCACTGGTGAGAACCCCCT | 141 | 2 and 3 | 62 | XM_533568 |
|  | L | CCTGATTCACACGGCGTAG |  | 3 |  |  |
| *B2M* | U | TCCTCATCCTCCTCGCT | 85 | 1 | 61 | XM_535458 |
|  | L | TTCTCTGCTGGGTGTCG |  | 2 |  |  |
| *HMBS* | U | TCACCATCGGAGCCATCT | 112 | 6 | 61 | XM_546491 |
|  | L | GTTCCCACCACGCTCTTCT |  | 6 and 7 |  |  |
| *RPL13A* | U | GCCGGAAGGTTGTAGTCGT | 87 | 3 | 61 | AJ388525 |
|  | L | GGAGGAAGGCCAGGTAATTC |  | 4 |  |  |
| *SDHA* | U | GCCTTGGATCTCTTGATGGA | 92 | 6 | 61 | XM_535807 |
|  | L | TTCTTGGCTCTTATGCGATG |  | 6 |  |  |
| *YWHAZ* | U | CGAAGTTGCTGCTGGTGA | 94 | 2 | 58 | XM_533072 |
|  | L | TTGCATTTCCTTTTTGCTGA |  | 2 and 3 |  |  |
